# Clinical Audit of Clozapine Prescribing Practice and Monitoring Process in an Australian Community Mental Health Service

**DOI:** 10.1192/bjo.2022.83

**Published:** 2022-06-20

**Authors:** Tharushi Fernando, Ritesh Bhandarkar, Graham Meadows

**Affiliations:** Monash Health, Melbourne, Australia

## Abstract

**Aims:**

Clozapine, a well-established treatment of choice for treatment-resistant schizophrenia is known to reduce suicidality, lessen the risk of tardive dyskinesia and reduce relapse risk. It contributes to a higher quality of life by reducing cognitive clouding. Patients taking Clozapine have improved social and work functioning. But Clozapine's significant side effects require regular, intense monitoring to minimize mortality and morbidity. To improve current practice of clozapine prescribing and monitoring, a systematic audit of service practices against guidelines of local hospital / Monash Health Clozapine patient management guidelines and the Royal Australian and New Zealand College of Psychiatrists (RANZCP) clinical practice guidelines will identify any deficits and inform measures to overcome them.

**Methods:**

An audit was conducted to compare the current clozapine prescribing practice and monitoring process compared with local hospital / Monash Health Clozapine patient management guidelines and RANZCP clinical practice guidelines among clozapine prescribed patients in an Australian community mental health service.

**Results:**

Medical records of thirty-three eligible adult patients on clozapine were audited. All the patients were prescribed dosages within the recommended daily clozapine range. Clozapine was used for appropriate indications (treatment of treatment resistant-schizophrenia or schizoaffective disorder). Of the 33 patients, clozapine level was subtherapeutic on 54.5% of patients. 54.5% of patients were on an adjunct psychotropic with clozapine. Aripiprazole and sodium valproate were used by eight patients each, and nine patients were identified using selective serotonin reuptake inhibitors. The most common side effect was hypersalivation (57.6%), followed by weight gain (39.4%), sedation (21.2%) and constipation (12.1%). Monthly weight monitoring, physical examination, medical officer monthly review and full blood examination, at 97% compliance met these standards. However, monitoring of Body Mass Index (BMI) (66.7%) and six-monthly consultant reviews (42.4%) showed poor compliance (<70%) with the standards. Most metabolic blood investigations were in moderate compliance (70–90%) except for relatively high compliance for lipid profile (90.1%). Monitoring cardiac functions by echocardiogram were only 75.8% met the standard.

**Conclusion:**

Most patients in this clinic receive recommended monthly monitoring practice but for BMI monitoring, six-monthly consultant review, most blood investigations and annual or 2 yearly echocardiogram findings indicated need for improvement. Polypharmacy of psychotropics increases the side effect burden and further increases the need to closely monitor the physical health and prescriptions of this cohort of patients. The next stage of this project will involve a codesign approach to developing a response to these findings that will be outlined here.

